# Supplementary material for: Single-molecule fluorescence microscopy reveals regulatory mechanisms of MYO7A-driven cargo transport in stereocilia of live inner ear hair cells
Source: Nat Commun. 2025 Sep 1;16:8149. doi: 10.1038/s41467-025-63102-0 (PMC12402077; doi:10.1038/s41467-025-63102-0)
Supplement: Supplementary file 1 — Supplementary Information [file 41467_2025_63102_MOESM1_ESM.pdf]

Supplementary Figures

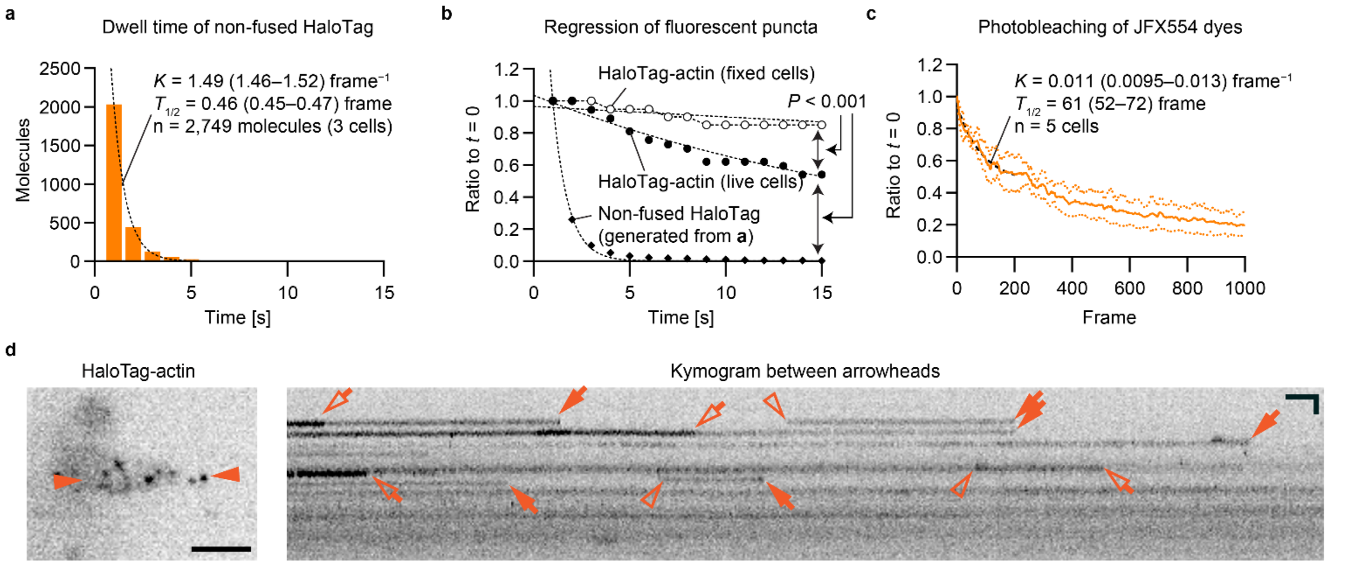

Supplementary Figure 1: Behaviors of non-fused HaloTag and HaloTag-actin molecules

a, Dwell-time distribution of non-fused HaloTag molecules in live-cell stereocilia. More than 70% of fluorescent puncta disappear after one frame. The rate constant (K) and half-life ($T_{1/2}$) are shown with 95% CIs. Time-lapse images are acquired every 1 s. **b**, Regression of HaloTag-actin fluorescent puncta from t = 0 in live cells (n = 37 from 6 cells) and fixed cells (n = 20 from 4 cells). The survival curve of non-fused HaloTag is generated from **a** and plotted for comparison. Fitting of one-phase decay models shows regression being slower in the order of non-fused HaloTag (K = 1.27 [95% CI: 1.23–1.31] s^{-1}, $T_{1/2}$ = 0.54 [0.52–0.56] s), HaloTag-actin in live cells (K = 0.049 [0.047–0.051] s^{-1}, $T_{1/2}$ = 13.9 [13.3–14.5] s) and HaloTag-actin in fixed cells (K = 0.0064 [0.0029–0.0099] s^{-1}, $T_{1/2}$ = 107 [69–231] s). The differences are statistically significant against a null hypothesis that one model explains all data using the extra-sum-of-squares F-test followed by the Bonferroni correction. Single-plane time-lapse, every 1 s. **c**, Photobleaching of JFX554 dyes conjugated to HaloTag-actin measured using the cuticular plate of fixed hair cells. The K and $T_{1/2}$ values are similar to those calculated for the regression of HaloTag-actin molecules in fixed cells (**b**). This photobleaching is apparent because some dyes switch to the dark state and do not decay[1]. Time-lapse images are acquired every 100 ms. SDs, dotted lines. **d**, Quantum behavior of JFX554 dyes conjugated to HaloTag-actin in fixed cells. Trajectories in kymograms suddenly terminate (arrows) or decrease their fluorescence intensity (open arrows). Some dyes recover from the dark state (open arrowheads). These quantum behaviors suggest that single fluorophores are detected in our methodology. Single-plane time-lapse, every 100 ms. Bars, 5 µm (cell image); 2 µm and 2 s (kymogram). Source data are provided as a Source Data file.

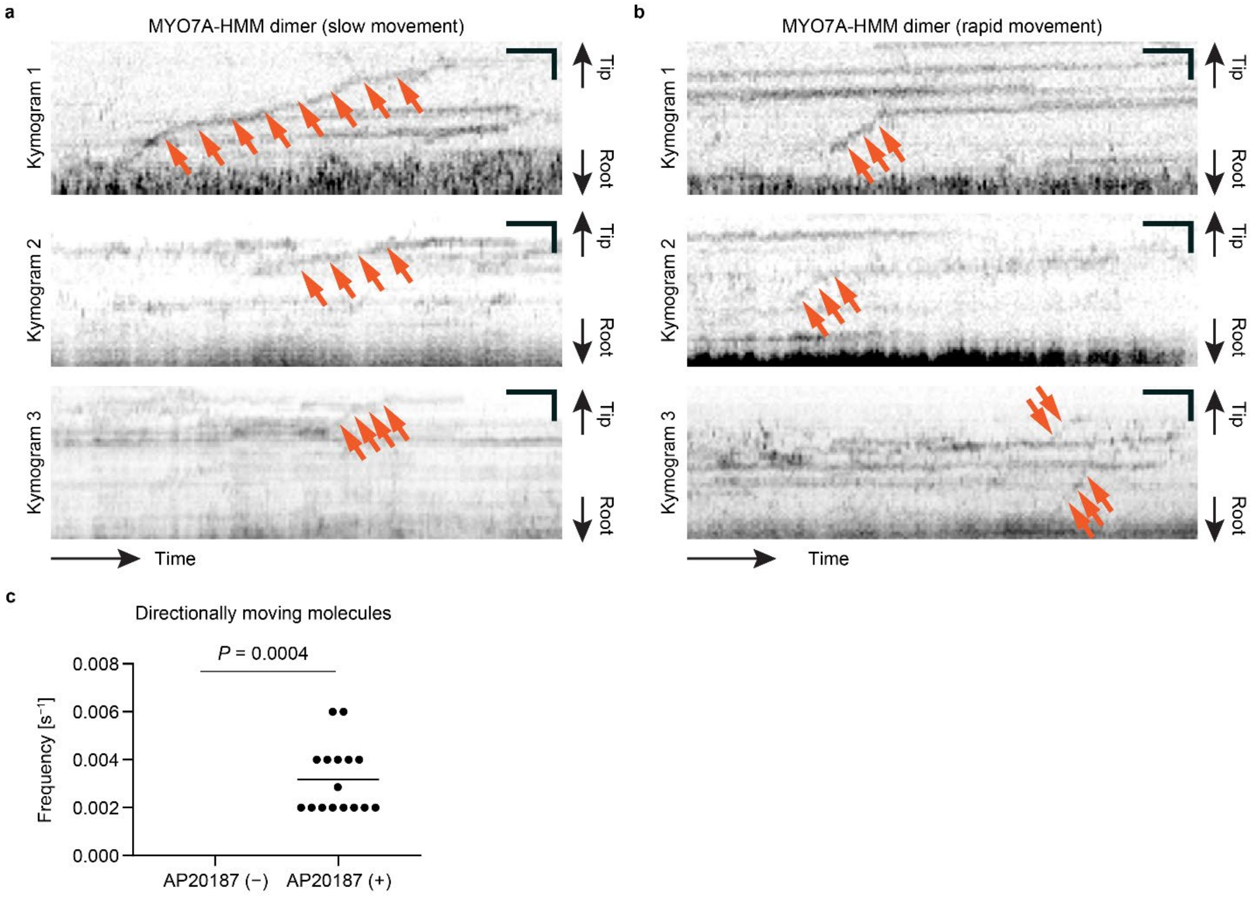

Supplementary Figure 2: Movement of MYO7A-HMM dimers in stereocilia

a and b, Representative kymograms of slow (**a**) and rapid (**b**) movement of HaloTag-MYO7A-HMM-FKBP dimers in cells treated with 200 nM AP20187. Trajectories are indicated by arrows. Single-plane time-lapse, every 1 s. Bars, 2 μm and 20 s. **c**, Semi-quantified frequency of directionally moving molecules compared between non-treated cells (0 s^{-1}, n = 4 images from 4 cells) and cells treated with 200 nM AP20187 (0.0031 ± 0.0014 s^{-1}, mean ± SD, n = 16 images from 11 cells). P-value by Student's t-test. Source data are provided as a Source Data file.

a Number of hair cells showing MYO7A accumulation at stereocilia tips

	Accumulation at the tips		
	Blob	Weakly	None
Full length MYO7A	*0	2	*7
MYO7A-RK/AA	*5	0	*1
MYO7A-ΔSH3-ΔM/F2	2	0	4

* P = 0.042; data from 3 independent experiments

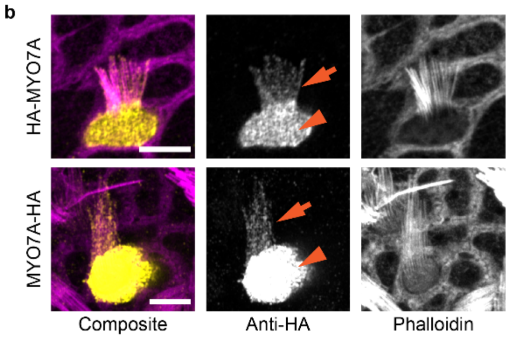

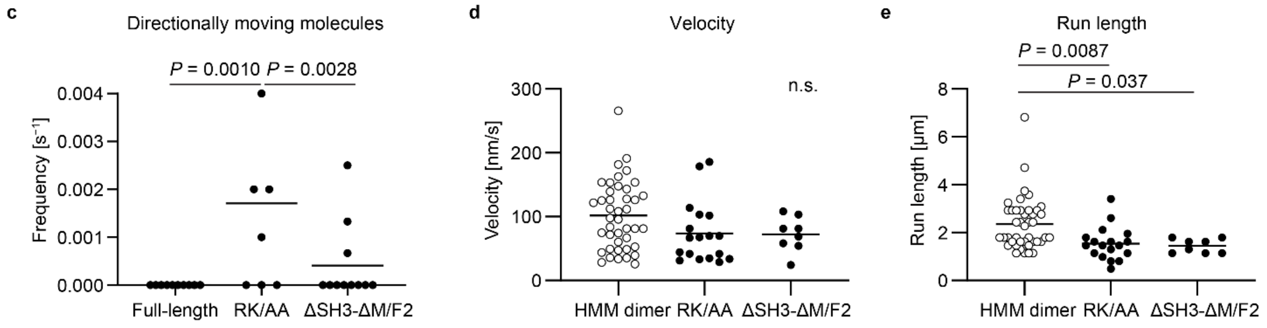

Supplementary Figure 3: Movement of constitutively active MYO7A mutants in stereocilia

a, Number of hair cells showing accumulation of HaloTag-fused MYO7A at stereocilia tips under confocal microscopy. Accumulation of MYO7A at the tips is classified into three levels: enlarged stereocilia tips (Blob), accumulation at the tips without morphological change (Weakly) and neither of these (None). Pairwise comparisons using Fisher's exact test show P = 0.042 between full-length MYO7A and MYO7A-RK/AA for the formation of protein blobs at stereocilia tips. **b**, Distribution of ectopically expressed full-length MYO7A, with an HA tag at the N-terminus (HA-MYO7A) or at the C-terminus (MYO7A-HA). Immunostaining for the HA-tag reveals that full-length MYO7A is diffusely distributed along stereocilia (arrows), similar to the distribution observed with HaloTag-fused full-length MYO7A. Signals from HA-MYO7A or MYO7A-HA are more intense in the cell body (arrowheads). Bar, 5 μm. **c**, Semi-quantified frequency of directionally moving molecules under single-molecule microscopy. MYO7A-RK/AA molecules move at a significantly higher frequency (0.0017 ± 0.0018 s^{-1}, mean ± SD, n = 8 images from 8 cells) than full-length MYO7A (0 s^{-1}, n = 10 images from 10 cells) and MYO7A-ΔSH3-ΔM/F2 (0.00040 ± 0.00081 s^{-1}, n = 11 images from 11 cells). One-way ANOVA shows P = 0.0073. Post-hoc test by Tukey. **d**, Velocity of directional movement compared with control MYO7A-HMM dimers (Fig. 2e). MYO7A-R/K and MYO7A-ΔSH3-ΔM/F2 move at 73 ± 47 nm/s (n = 18) and 72 ± 27 nm/s (n = 9), respectively, without significant difference against MYO7A-HMM dimers (101 ± 53 nm/s). P = 0.083 by one-way ANOVA. **e**, Run-lengths of directional movement compared with MYO7A-HMM dimers (Fig. 2f). The run lengths are slightly shorter for MYO7A-R/K (1.54 ± 0.69 μm, n = 18) and MYO7A-ΔSH3-ΔM/F2 (1.44 ± 0.28 μm, n = 8) than MYO7A-HMM dimers (2.3 ± 1.0 μm). One-way ANOVA shows P = 0.0027. Post-hoc test by Tukey. Source data are provided as a Source Data file.

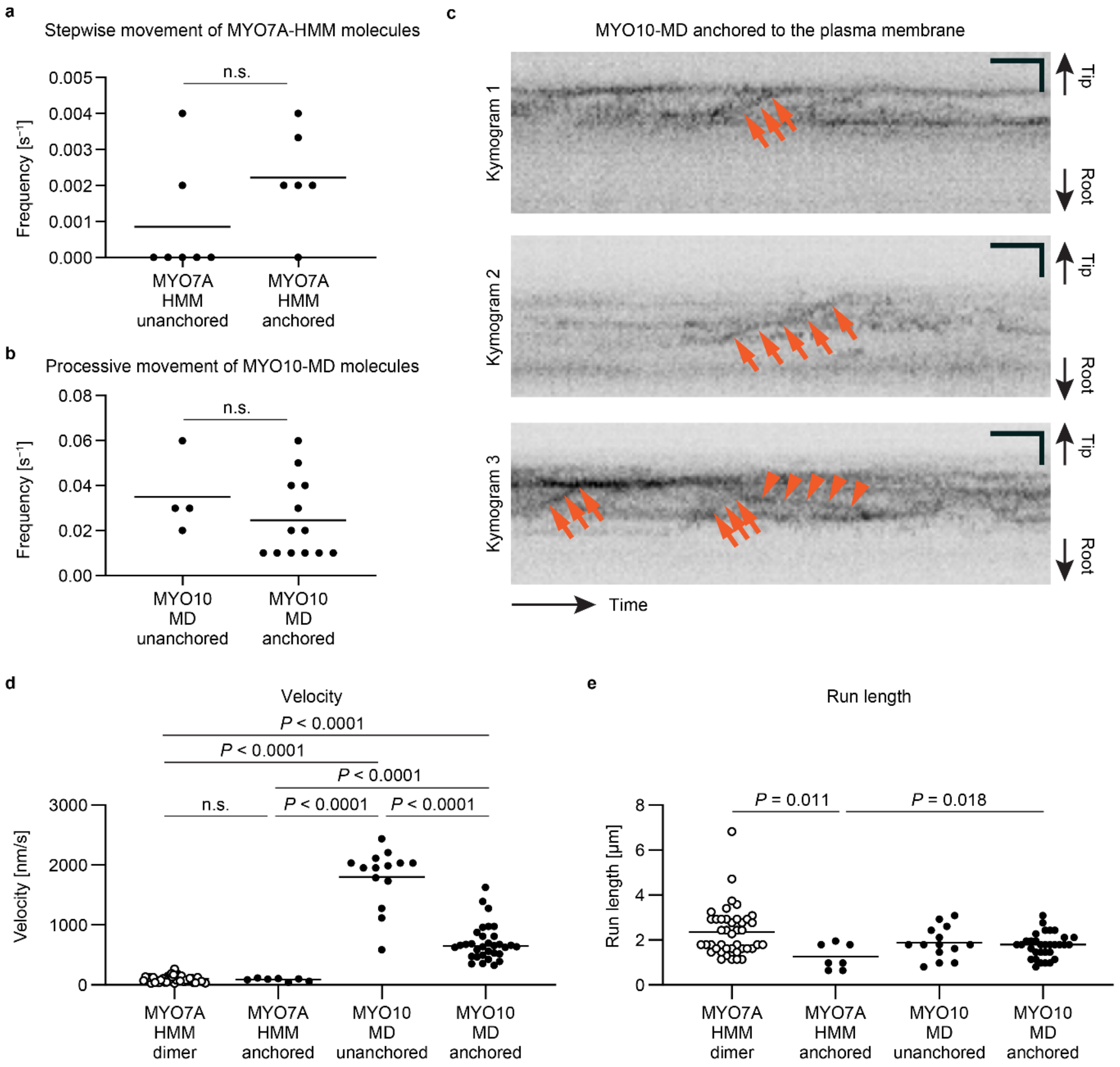

Supplementary Figure 4: Movement of MYO7A-HMM and MYO10-MD anchored to the plasma membrane

a, Semi-quantified frequency of directionally moving unanchored MYO7A-HMM (0.00085 ± 0.0015 s^{-1}, mean ± SD, n = 7 images from 4 cells) and membrane-anchored MYO7A-HMM (0.0022 ± 0.0013 s^{-1}, n = 6 from 4 cells). Membrane-anchored MYO7A-HMM moves slightly at a higher frequency but without significant differences by Student's *t*-test. **b**, Semi-quantified frequency of directionally moving unanchored MYO10-MD (0.035 ± 0.017 s^{-1}, n = 4 from 3 cells) and membrane-anchored MYO10-MD (0.024 ± 0.017 s^{-1}, n = 13 from 3 cells). No significant difference is detected by Student's *t*-test. **c**, Representative kymograms showing slow movement of membrane-anchored MYO10-MD molecules. Trajectories are continuous and consistent with processive movement (arrows). Retrograde movement is also observed (arrowheads). Single-plane time-lapse, every 100 ms. Bars, 2 µm and 2 s. **d**, Velocity of directionally moving MYO7A-HMM dimers (101 ± 53 nm/s, data from Fig. 2e), membrane-anchored MYO7A-HMM (88 ± 27 nm/s, n = 7), unanchored MYO10-MD (1,800 ± 490 nm/s, n = 14) and membrane-anchored MYO10-MD (701 ± 297 nm/s, n = 32). MYO10-MD moves more slowly when anchored to the plasma membrane. Unanchored and membrane-anchored MYO10-MD molecules move significantly faster than MYO7A-HMM

dimers and membrane-anchored MYO7A-HMM. One-way ANOVA shows $P < 0.0001$. Post-hoc test by Tukey. **e**, Run-lengths of directionally moving MYO7A-HMM dimers (2.3 ± 1.0 μm, Fig. 2f), membrane-anchored MYO7A-HMM (1.2 ± 0.56 μm, $n = 7$), unanchored MYO10-MD (1.8 ± 0.7 μm, $n = 14$) and membrane-anchored MYO10-MD (1.7 ± 0.54 μm, $n = 32$). Membrane-anchored MYO7A-HMM runs over slightly shorter distances than MYO7A-HMM dimers and membrane-anchored MYO10-MD. One-way ANOVA shows $P = 0.0023$ although the differences are small. Post-hoc test by Tukey. Source data are provided as a Source Data file.

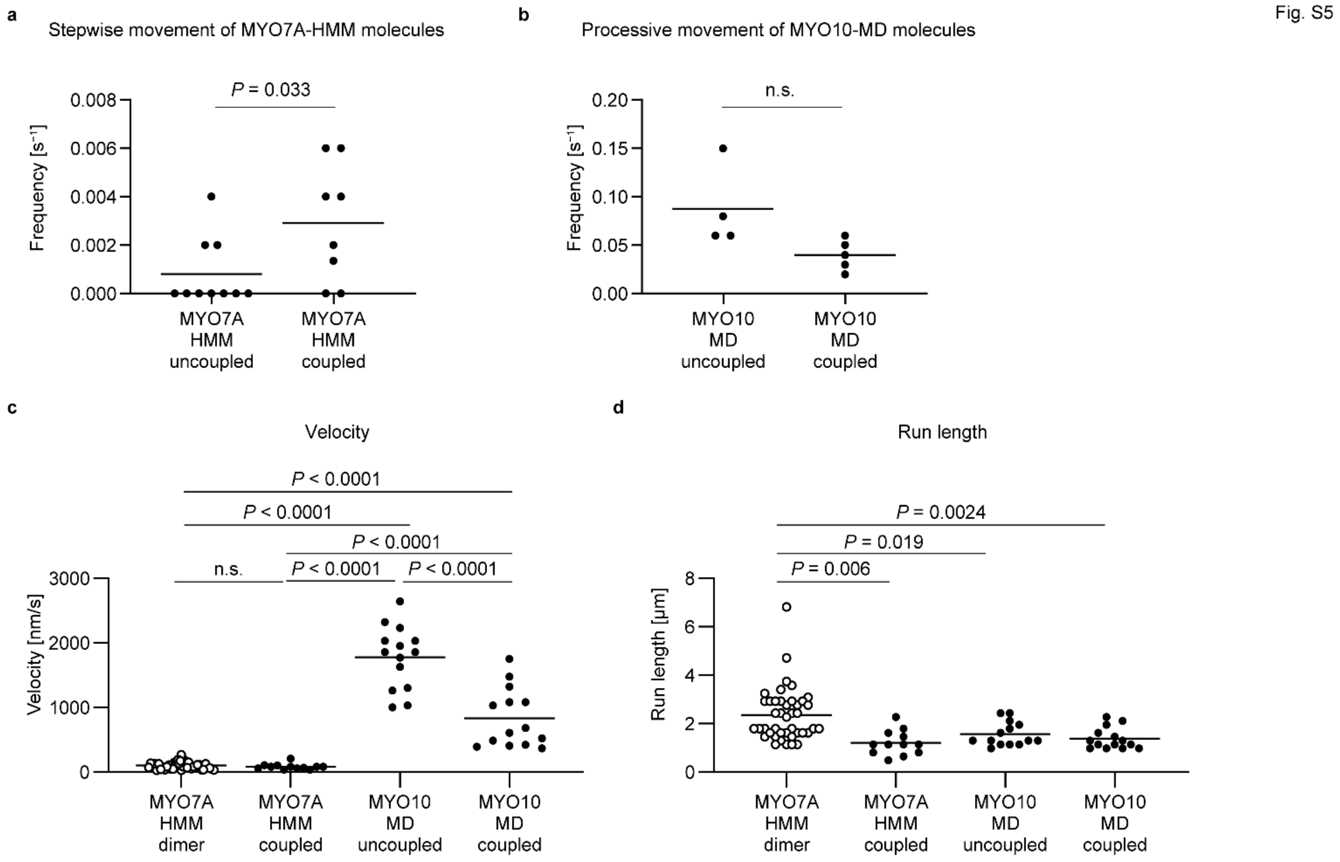

Supplementary Figure 5: Movement of MYO7A-HMM and MYO10-MD coupled with a harmonin b fragment

a, Semi-quantified frequency of directionally moving uncoupled MYO7A-HMM (0.00080 ± 0.0013 s^{-1}, mean ± SD, *n* = 10 images from 4 cells) and MYO7A-HMM coupled with a harmonin b fragment (0.0032 ± 0.0025 s^{-1}, *n* = 9 images from 7 cells). *P*-value by Student's *t*-test. **b**, Semi-quantified frequency of directionally moving uncoupled MYO10-MD (0.087 ± 0.042 s^{-1}, *n* = 4 images from 4 cells) and MYO10-MD coupled with a harmonin b fragment (0.040 ± 0.015 s^{-1}, *n* = 5 images from 3 cells). No significant difference is detected by Student's *t*-test. **c**, Velocity of directionally moving MYO7A-HMM dimers (101 ± 53 nm/s, Fig. 2e), MYO7A-HMM coupled with a harmonin b fragment (84 ± 44 nm/s, *n* = 12), uncoupled MYO10-MD (1,780 ± 487 nm/s, *n* = 14) and MYO10-MD coupled with a harmonin b fragment (831 ± 456 nm/s, *n* = 14). MYO10-MD moves more slowly when coupled with a harmonin b fragment. Uncoupled MYO10-MD and MYO10-MD coupled with a harmonin b fragment move significantly faster than MYO7A-HMM dimers and MYO7A-HMM coupled with a harmonin b fragment. One-way ANOVA shows *P* < 0.0001. Post-hoc test by Tukey. **d**, Run-lengths of directionally moving MYO7A-HMM dimers (2.3 ± 1.0 µm, Fig. 2f), MYO7A-HMM coupled with a harmonin b fragment (1.20 ± 0.51 µm, *n* = 12), uncoupled MYO10-MD (1.56 ± 0.49 µm, *n* = 14) and MYO10-MD coupled with a harmonin b fragment (1.38 ± 0.44 µm, *n* = 14). MYO7A-HMM dimers move over longer distances than MYO7A-HMM coupled with a harmonin b fragment, uncoupled MYO10-MD and MYO10-MD coupled with a harmonin b fragment. One-way ANOVA shows *P* < 0.0001. Post-hoc test by Tukey. Source data are provided as a Source Data file.

Supplementary Movies

Supplementary Movie 1: Time-lapse images of non-fused HaloTag

Vestibular hair cell (P2) expressing non-fused HaloTag and imaged as a control for diffusing proteins. Most fluorescent puncta disappear after one frame. JFX554, 0.1 nM. Single-plane time-lapse, every 1 s. Exposure, 100 ms at 0.2 kW/cm^2. Bar, 5 µm.

Supplementary Movie 2: Time-lapse images of HaloTag-actin

Vestibular hair cell (P2) expressing HaloTag-actin and imaged as a control for proteins stably bound to the F-actin core. Most of the fluorescent puncta remain in the same location and disappear suddenly due to photobleaching or transition to the dark state (representatively indicated by magenta circles). JFX554, 0.01 nM. Single-plane time-lapse, every 1 s. Exposure, 100 ms at 0.2 kW/cm^2. Bar, 5 µm.

Supplementary Movie 3: Time-lapse images of HaloTag-MYO7A-HMM-FKBP with AP20187 treatment

Vestibular hair cell (P2) expressing HaloTag-MYO7A-HMM-FKBP and imaged after adding 200 nM AP20187 to the culture medium. Molecules showing directional and processive movement are indicated by magenta circles. JFX554, 0.3 nM. Single-plane time-lapse, every 1 s. Exposure, 100 ms at 0.2 kW/cm^2. Bar, 5 µm.

Supplementary Movie 4: Time-lapse images of HaloTag-MYO7A-HMM-FKBP without AP20187 treatment

Vestibular hair cell (P2) expressing HaloTag-MYO7A-HMM-FKBP and imaged without adding AP21087 to the culture medium. No processive movement is observed. Circles indicate molecules showing stepwise movement toward stereocilia tips. JFX554, 0.3 nM. Single-plane time-lapse, every 1 s. Exposure, 100 ms at 0.2 kW/cm^2. Bar, 5 µm.

Supplementary Movie 5: Time-lapse images of HaloTag-MYO7A-RK/AA

Vestibular hair cell (P2) expressing HaloTag-MYO7A-RK/AA, which has two missense mutations (p.R2127A and p.K2130A) disabling autoinhibition of the motor domain. Imaged every 1 s by single-plane time-lapse acquisition. Three molecules showing directional movement are indicated by magenta circles. JFX554, 0.3 nM. Exposure, 100 ms at 0.2 kW/cm^2. Bar, 5 µm.

Supplementary Movie 6: Time-lapse images of HaloTag-MYO7A-ΔSH3-ΔM/F2

Vestibular hair cell (P2) expressing HaloTag-MYO7A-ΔSH3-ΔM/F2, whose tail is truncated to disable autoinhibition of the motor domain. Imaged every 1 s by single-plane time-lapse acquisition. A molecule showing directional movement is indicated by magenta circles. JFX554, 0.3 nM. Exposure, 100 ms at 0.2 kW/cm^2. Bar, 5 µm.

Supplementary Movie 7: Time-lapse images of membrane-anchored HaloTag-MYO7A-HMM-FRB

Vestibular hair cell (P2) co-expressing HaloTag-MYO7A-HMM-FRB and IL2Rα-EGFP-FKBP. The cell is treated with 500 nM AP21987 to anchor MYO7A-HMM to the plasma membrane. Molecules showing stepwise, directional movement toward stereocilia tips are indicated by magenta circles. Single-plane time-lapse, every 0.3 s. JFX554, 0.3 nM. Exposure, 100 ms at 0.2 kW/cm^2. Bar, 5 μm.

Supplementary Movie 8: Time-lapse images of HaloTag-MYO10-MD-FRB before membrane anchoring

Vestibular hair cell (P2) co-expressing HaloTag-MYO10-MD-FRB and IL2Rα-EGFP-FKBP. The cell is imaged without AP21987 treatment. A small number of molecules show rapid directional and processive movement toward stereocilia tips (magenta circles). Single-plane images are acquired every 100 ms. JFX554, 0.3 nM. Exposure, 100 ms at 0.2 kW/cm^2. Bar, 5 μm.

Supplementary Movie 9: Time-lapse images of HaloTag-MYO10-MD-FRB after membrane anchoring

Vestibular hair cell (P2) co-expressing HaloTag-MYO10-MD-FRB and IL2Rα-EGFP-FKBP. The cell is treated with 500 nM AP21987 to anchor MYO10-MD to the plasma membrane. Molecules showing slow processive movement are indicated by magenta circles. Single-plane images are acquired every 100 ms. JFX554, 0.3 nM. Exposure, 100 ms at 0.2 kW/cm^2. Bar, 5 μm.

Supplementary Movie 10: Time-lapse images of HaloTag-MYO7A-HMM-FKBP coupled with a harmonin b fragment

Vestibular hair cell (P2) co-expressing HaloTag-MYO7A-HMM-FKBP and FRB-DFCR-EGFP. The cell is treated with 500 nM AP21987 to tether the MYO7A-HMM C-terminus to F-actin. Molecules showing stepwise directional movement are indicated by magenta circles. Single-plane time-lapse, every 1 s. JFX554, 0.3 nM. Exposure, 100 ms at 0.2 kW/cm^2. Bar, 5 μm.

Supplementary Movie 11: Time-lapse images of HaloTag-MYO10-MD-FKBP coupled with a harmonin b fragment

Vestibular hair cell (P2) co-expressing HaloTag-MYO10-MD-FKBP and FRB-DFCR-EGFP. The cell is treated with 500 nM AP21987 to tether the MYO10-MD C-terminus to F-actin. Molecules showing slow directional movement are indicated by magenta circles. Single-plane time-lapse, every 100 ms. JFX554, 0.3 nM. Exposure, 100 ms at 0.2 kW/cm^2. Bar, 5 μm.

References

1 Endesfelder, U. & Heilemann, M. Direct stochastic optical reconstruction microscopy (dSTORM). *Methods Mol Biol* **1251**, 263-276 (2015). https://doi.org/10.1007/978-1-4939-2080-8_14
