# Supplementary material for: Single-molecule fluorescence microscopy reveals regulatory mechanisms of MYO7A-driven cargo transport in stereocilia of live inner ear hair cells
Source: Nat Commun. 2025 Sep 1;16:8149. doi: 10.1038/s41467-025-63102-0 (PMC12402077; doi:10.1038/s41467-025-63102-0)
Supplement: Supplementary file 13 — Description of additional supplementary files [file 41467_2025_63102_MOESM13_ESM.pdf]

Description of Additional Supplementary Files

Supplementary Movie 1: Time-lapse images of non-fused HaloTag

Vestibular hair cell (P2) expressing non-fused HaloTag and imaged as a control for diffusing proteins. Most fluorescent puncta disappear after one frame. JFX554, 0.1 nM. Single-plane time-lapse, every 1 s. Exposure, 100 ms at 0.2 kW/cm^2. Bar, 5 µm.

Supplementary Movie 2: Time-lapse images of HaloTag-actin

Vestibular hair cell (P2) expressing HaloTag-actin and imaged as a control for proteins stably bound to the F-actin core. Most of the fluorescent puncta remain in the same location and disappear suddenly due to photobleaching or transition to the dark state (representatively indicated by magenta circles). JFX554, 0.01 nM. Single-plane time-lapse, every 1 s. Exposure, 100 ms at 0.2 kW/cm^2. Bar, 5 µm.

Supplementary Movie 3: Time-lapse images of HaloTag-MYO7A-HMM-FKBP with AP20187 treatment

Vestibular hair cell (P2) expressing HaloTag-MYO7A-HMM-FKBP and imaged after adding 200 nM AP20187 to the culture medium. Molecules showing directional and processive movement are indicated by magenta circles. JFX554, 0.3 nM. Single-plane time-lapse, every 1 s. Exposure, 100 ms at 0.2 kW/cm^2. Bar, 5 µm.

Supplementary Movie 4: Time-lapse images of HaloTag-MYO7A-HMM-FKBP without AP20187 treatment

Vestibular hair cell (P2) expressing HaloTag-MYO7A-HMM-FKBP and imaged without adding AP21087 to the culture medium. No processive movement is observed. Circles indicate molecules showing stepwise movement toward stereocilia tips. JFX554, 0.3 nM. Single-plane time-lapse, every 1 s. Exposure, 100 ms at 0.2 kW/cm^2. Bar, 5 µm.

Supplementary Movie 5: Time-lapse images of HaloTag-MYO7A-RK/AA

Vestibular hair cell (P2) expressing HaloTag-MYO7A-RK/AA, which has two missense mutations (p.R2127A and p.K2130A) disabling autoinhibition of the motor domain. Imaged every 1 s by single-plane time-lapse acquisition. Three molecules showing directional movement are indicated by magenta circles. JFX554, 0.3 nM. Exposure, 100 ms at 0.2 kW/cm^2. Bar, 5 µm.

Supplementary Movie 6: Time-lapse images of HaloTag-MYO7A-ΔSH3-ΔM/F2

Vestibular hair cell (P2) expressing HaloTag-MYO7A-ΔSH3-ΔM/F2, whose tail is truncated to disable autoinhibition of the motor domain. Imaged every 1 s by single-plane time-lapse

acquisition. A molecule showing directional movement is indicated by magenta circles. JFX554, 0.3 nM. Exposure, 100 ms at 0.2 kW/cm^2. Bar, 5 µm. 8

Supplementary Movie 7: Time-lapse images of membrane-anchored HaloTag-MYO7A-HMM-FRB

Vestibular hair cell (P2) co-expressing HaloTag-MYO7A-HMM-FRB and IL2Rα-EGFP-FKBP. The cell is treated with 500 nM AP21987 to anchor MYO7A-HMM to the plasma membrane. Molecules showing stepwise, directional movement toward stereocilia tips are indicated by magenta circles. Single-plane time-lapse, every 1 s. JFX554, 0.3 nM. Exposure, 100 ms at 0.2 kW/cm^2. Bar, 5 µm.

Supplementary Movie 8: Time-lapse images of HaloTag-MYO10-MD-FRB before membrane anchoring

Vestibular hair cell (P2) co-expressing HaloTag-MYO10-MD-FRB and IL2Rα-EGFP-FKBP. The cell is imaged without AP21987 treatment. A small number of molecules show rapid directional and processive movement toward stereocilia tips (magenta circles). Single-plane images are acquired every 100 ms. JFX554, 0.3 nM. Exposure, 100 ms at 0.2 kW/cm^2. Bar, 5 µm.

Supplementary Movie 9: Time-lapse images of HaloTag-MYO10-MD-FRB after membrane anchoring

Vestibular hair cell (P2) co-expressing HaloTag-MYO10-MD-FRB and IL2Rα-EGFP-FKBP. The cell is treated with 500 nM AP21987 to anchor MYO10-MD to the plasma membrane. Molecules showing slow processive movement are indicated by magenta circles. Single-plane images are acquired every 100 ms. JFX554, 0.3 nM. Exposure, 100 ms at 0.2 kW/cm^2. Bar, 5 µm.

Supplementary Movie 10: Time-lapse images of HaloTag-MYO7A-HMM-FKBP coupled with a harmonin b fragment

Vestibular hair cell (P2) co-expressing HaloTag-MYO7A-HMM-FKBP and FRB-DFCR-EGFP. The cell is treated with 500 nM AP21987 to tether the MYO7A-HMM C-terminus to F-actin. Molecules showing stepwise directional movement are indicated by magenta circles. Single-plane time-lapse, every 1 s. JFX554, 0.3 nM. Exposure, 100 ms at 0.2 kW/cm^2. Bar, 5 µm.

Supplementary Movie 11: Time-lapse images of HaloTag-MYO10-MD-FKBP coupled with a harmonin b fragment

Vestibular hair cell (P2) co-expressing HaloTag-MYO10-MD-FKBP and FRB-DFCR-EGFP. The cell is treated with 500 nM AP21987 to tether the MYO10-MD C-terminus to F-actin. Molecules showing slow directional movement are indicated by magenta circles. Single-plane time-lapse, every 100 ms. JFX554, 0.3 nM. Exposure, 100 ms at 0.2 kW/cm^2. Bar, 5 µm.
