# Supplementary material for: Single-molecule fluorescence microscopy reveals regulatory mechanisms of MYO7A-driven cargo transport in stereocilia of live inner ear hair cells
Source: Nat Commun. 2025 Sep 1;16:8149. doi: 10.1038/s41467-025-63102-0 (PMC12402077; doi:10.1038/s41467-025-63102-0)
Supplement: Supplementary file 14 — Reporting Summary [file 41467_2025_63102_MOESM14_ESM.pdf]

nature portfolio

Corresponding author(s): Takushi Miyoshi

Last updated by author(s): Jul 19, 2025

Reporting Summary

Nature Portfolio wishes to improve the reproducibility of the work that we publish. This form provides structure for consistency and transparency in reporting. For further information on Nature Portfolio policies, see our Editorial Policies and the Editorial Policy Checklist.

Statistics

For all statistical analyses, confirm that the following items are present in the figure legend, table legend, main text, or Methods section.

n/a	Confirmed	
☐	☒	The exact sample size (*n*) for each experimental group/condition, given as a discrete number and unit of measurement
☐	☒	A statement on whether measurements were taken from distinct samples or whether the same sample was measured repeatedly
☐	☒	The statistical test(s) used AND whether they are one- or two-sided<br>*Only common tests should be described solely by name; describe more complex techniques in the Methods section.*
☒	☐	A description of all covariates tested
☐	☒	A description of any assumptions or corrections, such as tests of normality and adjustment for multiple comparisons
☐	☒	A full description of the statistical parameters including central tendency (e.g. means) or other basic estimates (e.g. regression coefficient) AND variation (e.g. standard deviation) or associated estimates of uncertainty (e.g. confidence intervals)
☐	☒	For null hypothesis testing, the test statistic (e.g. *F*, *t*, *r*) with confidence intervals, effect sizes, degrees of freedom and *P* value noted<br>*Give P values as exact values whenever suitable.*
☒	☐	For Bayesian analysis, information on the choice of priors and Markov chain Monte Carlo settings
☒	☐	For hierarchical and complex designs, identification of the appropriate level for tests and full reporting of outcomes
☒	☐	Estimates of effect sizes (e.g. Cohen's *d*, Pearson's *r*), indicating how they were calculated

Our web collection on statistics for biologists contains articles on many of the points above.

Software and code

Policy information about availability of computer code

Data collection	Single-molecule microscopy data were collected using Micro-Manager (https://micro-manager.org/) plugin for ImageJ. Confocal microscopy data were collected using proprietary Zeiss ZEN black.
Data analysis	Image data were analyzed using ImageJ, Python and its open-source libraries (Scipy and scikit-image). Custom Python scripts used in this study have been archived on Zenodo and assigned the following DOIs: momomagick (https://doi.org/10.5281/zenodo.16503793) and momotrack (https://doi.org/10.5281/zenodo.16503757). For continued development and the latest updates, the code is also available at the first author's GitHub repository (https://github.com/takushim). Statistical analyses were performed using GraphPad Prism and R (https://cran.r-project.org/).

For manuscripts utilizing custom algorithms or software that are central to the research but not yet described in published literature, software must be made available to editors and reviewers. We strongly encourage code deposition in a community repository (e.g. GitHub). See the Nature Portfolio guidelines for submitting code & software for further information.

Data

Policy information about availability of data

All manuscripts must include a data availability statement. This statement should provide the following information, where applicable:

- Accession codes, unique identifiers, or web links for publicly available datasets
- A description of any restrictions on data availability
- For clinical datasets or third party data, please ensure that the statement adheres to our policy

The numerical data underlying all graphs in this study are provided in the Source Data file. Representative images from single-molecule imaging experiments are shown in the main figures and are also available as Supplementary Movies. Raw single-molecule and confocal imaging data have been deposited in Zenodo (https://10.5281/zenodo.16233094). The custom plasmids used in this study will be distributed via Addgene. Image acquisition and analysis procedures are described in the Methods section.

Research involving human participants, their data, or biological material

Policy information about studies with human participants or human data. See also policy information about sex, gender (identity/presentation), and sexual orientation and race, ethnicity and racism.

Reporting on sex and gender	N/A
Reporting on race, ethnicity, or other socially relevant groupings	N/A
Population characteristics	N/A
Recruitment	N/A
Ethics oversight	N/A

Note that full information on the approval of the study protocol must also be provided in the manuscript.

Field-specific reporting

Please select the one below that is the best fit for your research. If you are not sure, read the appropriate sections before making your selection.

☒ Life sciences ☐ Behavioural & social sciences ☐ Ecological, evolutionary & environmental sciences

For a reference copy of the document with all sections, see nature.com/documents/nr-reporting-summary-flat.pdf

Life sciences study design

All studies must disclose on these points even when the disclosure is negative.

Sample size	Power analyses were not available beforehand because this is the first study performing single-molecule microscopy in live hair cell stereocilia. Specifically, effect sizes were difficult to predict. Thus, we performed experiments at least three different cells from three different inner-ear sensory epithelia (or more cells whenever available) as a pilot study. For measurements of velocities and run lengths, we used the data from pilot studies because power analyses indicated sufficient sample sizes (e.g., actual power close to 1.0 for Fig. S3c). For the frequency of directionally-moving molecules, we used the results of pilot studies when statistical analyses yielded significant differences (e.g., P = 0.0004 of Fig. S2c). However, there were some pilot studies suggesting that our intervention would not change the frequency of directionally-moving molecules significantly (e.g., Fig. 4a and 4b, final data shown). For these data, power analyses indicated that each dataset required more than 60 cells to prevent Type-II errors. Considering that these data were semi-quantification and not relevant to the conclusion of our study, we supplemented these data using a few additional cells and confirmed that the results would not change.
Data exclusions	Inner ear hair cells were excluded from analyses when stereocilia were lost or severely deformed. For single-molecule analyses, molecules moving back and forth in a stereocilium were presumed to be in random molecular motion and removed from tracking.
Replication	All single-molecule microscopy experiments were performed using at least three different cells from three different inner-ear sensory epithelia.
Randomization	Mouse pups were randomly selected to prepare explant cultures of sensory epithelia. Then, the sensory epithelia were randomly selected for drug treatments.
Blinding	Time-lapse images were analyzed by a different researcher who does not know the details of experiments.

Reporting for specific materials, systems and methods

We require information from authors about some types of materials, experimental systems and methods used in many studies. Here, indicate whether each material, system or method listed is relevant to your study. If you are not sure if a list item applies to your research, read the appropriate section before selecting a response.

Materials & experimental systems

n/a	Involved in the study
☐	☒ Antibodies
☒	☐ Eukaryotic cell lines
☒	☐ Palaeontology and archaeology
☐	☒ Animals and other organisms
☒	☐ Clinical data
☒	☐ Dual use research of concern
☒	☐ Plants

Methods

n/a	Involved in the study
☒	☐ ChIP-seq
☒	☐ Flow cytometry
☒	☐ MRI-based neuroimaging

Antibodies

Antibodies used	HA-Tag (C29F4) Rabbit mAb #3724 (Cell Signaling Technology), Alexa Fluor 568-conjugated goat anti-rabbit IgG (H+L) antibody (A-11036, Thermo Fisher Scientific)
Validation	Antibodies were validated using HeLa cells expressing HA-tagged β-actin.

Animals and other research organisms

Policy information about studies involving animals; ARRIVE guidelines recommended for reporting animal research, and Sex and Gender in Research

Laboratory animals	C57BL/6J mice
Wild animals	Not used
Reporting on sex	We did not determine the sex of mice because it is unlikely that the sex of mice affects the trafficking of myosin molecules in stereocilia. However, tail samples were collected for all mice to determine their sex using PCR upon request.
Field-collected samples	N/A
Ethics oversight	All animal experiments were performed in accordance with the National Institutes of Health Guidelines for the Care and Use of Laboratory Animals and approved by the Animal Care and Use Committees at the NIH (No. 1263 to TBF) and SIU (No. 23-027 to TM).

Note that full information on the approval of the study protocol must also be provided in the manuscript.

Plants

Seed stocks	N/A
Novel plant genotypes	N/A
Authentication	N/A
